# Does lidocaine reduce pain intensity during corticosteroid injection? A double-blind randomized controlled equivalence trial

**DOI:** 10.1177/17531934241245036

**Published:** 2024-04-20

**Authors:** Teun Teunis, George E. Sayegh, Amir Fatehi, David Ring, Gregg Vagner, Lee Reichel

**Affiliations:** Department of Surgery and Perioperative Care Dell Medical School, Austin, TX, USA

**Keywords:** Bayesian trial design, corticosteroid injection, hand and wrist, lidocaine, pain intensity

## Abstract

Of the strategies considered to limit the discomfort of corticosteroid injection, one is to inject without lidocaine to reduce the total volume and avoid acidity. In a Bayesian trial, adults receiving corticosteroid injections were randomized to receive 0.5 mL of triamcinolone with or without 0.5 mL of lidocaine. Serial analysis was performed until a 95% probability of presence or absence of a 1.0-point difference in pain intensity on the 0–10 Numerical Rating Scale was reached. Injections with lidocaine were associated with a median of 2.4-point lower pain intensity during injection with a 95% probability of at least a 1-point reduction. The 95% probability was confirmed in 90% of the repeated analysis (36/40). Lidocaine is associated with lower immediate pain intensity during corticosteroid injection for hand and wrist conditions.

**Level of evidence:** I

## Introduction

Efforts to limit the pain of corticosteroid injection in the hand and wrist have included buffering of the lidocaine with sodium bicarbonate (Lee et al., 2013), image-guidance (Saha et al., 2023) and the application of ice after injection (An et al., 2016). The evidence regarding the association of injection pH with reduced pain intensity is inconclusive, with some studies finding that lower pain intensity buffering reduces injection acidity (Bartfield et al., 1993) and others showing no differences with buffering (Lewis et al., 2005).

It is possible that injecting corticosteroid without any lidocaine would be more comfortable than with lidocaine. Lidocaine is acidic and adds to the volume of the injection (Heise et al., 2014; Zijlstra et al., 2018). In two double-blinded randomized control trials, injection volume was found to be associated with injection-related pain intensity (Heise et al., 2014; Jørgensen et al., 1996). A randomized double-blind controlled trial found that dermatologic intralesional corticosteroid injections in the arm were more painful with co-injection of lidocaine and epinephrine (Zakria et al., 2022). In another randomized double-blinded control trial, participants who received corticosteroid injections with lidocaine and epinephrine experienced greater pain intensity than participants who only received corticosteroid injections for trigger finger (Patrinely et al., 2021).

Given that the lidocaine has the added downside of temporary loss of sensibility, we were interested in testing this in our practice and considering a change. We performed a Bayesian clinical trial (Teunis et al., 2023) among people receiving a corticosteroid injection for hand or wrist conditions. The aim of this study was to assess the difference in pain intensity during injection (directly assessed after the injection) between patients receiving corticosteroid injections with and without lidocaine for any hand or wrist injection. The secondary aims were to establish whether there was a difference in pain intensity at 5 minutes and at 4 hours after the injection.

## Methods

### Study design

The study was registered in the ClinicalTrials.gov registry (identifier NCT06188221). Ethical approval for this study was obtained (protocol number 2019-05-0094). The design and reporting format conform to CONSORT guidelines. After approval from the institutional review board, we prospectively included adult (age 18–89 years) English-speaking patients offered a steroid injection between 7 January 2020 and 17 May 2023. The long study interval was due to a pause during the COVID-19 epidemic. This was a double-blinded randomized controlled trial that employed a Bayesian trial design. The Bayesian trial design utilizes Bayes theory to generate probabilities based on prior observed data. Bayesian trial design analyses the data collected at regular intervals and terminates the enrolment when the target probability is achieved or when the target probability is deemed unobtainable with the resources available. This can reduce the number of data points required when compared with frequentist models.

Patients were recruited from the urban outpatient clinics of three hand surgeons. We excluded non–English-speaking patients and those unable to provide written and verbal consent. Patients indicated that they were willing to participate before the injection. Participants were randomized to receive an injection of 0.5 mL (20 mg) of triamcinolone either with or without 0.5 mL of lidocaine (total 1 mL). A 1 mL syringe and a 27 gauge needle were used. Randomization was performed for each participant using the random number generator function in Excel. To achieve a double-blinded trial (surgeon and participant), the medical assistant who prepares the injection covered the syringe with tape to hide the volume. After the injection, participants filled out all relevant measures on tablets using HIPAA-compliant REDCap, assisted by a research assistant not involved in their care. Patients were asked to complete a survey on REDCap in the following order: demographics; prior steroid injection; pain intensity; Jefferson Scale of Patient Perceptions of Physician Empathy (JSPPPE); 4-item Pain Catastrophizing Scale (PCS-4); 2-item Patient Health Questionnaire (PHQ-2) and a 2-item Pain Self-efficacy Questionnaire (PSEQ-2).

### Study population

We initially planned a frequentist analysis for equivalence trials, necessitating 118 participants. Recognizing advantages to a Bayesian trial design, we transitioned after 24 patients (Teunis et al., 2023). Bayesian trial design concludes when the target probability is reached or when achieving the target probability is considered unattainable with the available resources. We reached our endpoint (95% probability of 1.0-point difference in pain intensity) at 39 participants: 22 (56%) in the corticosteroid group and 17 (44%) in the lidocaine with corticosteroid group (Table 1). In total, 32 (82%) participants received an injection for trigger digit, 5 (13%) received an injection for trapeziometacarpal arthritis and 2 (5%) received an injection for de Quervain syndrome (Table 1).

**Table 1.** Patient characteristics.

	Complete cohort	Steroid injection	Steroid injection with lidocaine
Participants	39	22	17
Age (years)	61 (53–71)	55 (51–61)	70 (65–74)
Men	17 (44)	7 (32)	10 (59)
JSPPPE	34 (31–35)	34 (27–35)	34 (33–35)
PCS	4 (2–8)	6 (2–9)	3 (2–4)
PHQ	0 (0–1)	0 (0–1)	0 (0–1)
PSEQ	10 (9–12)	10 (9–12)	10 (9–12)
Injection site			
A1 pulley (palmer)	32 (82)	17 (77)	15 (88)
1st extensor compartment (radial)	5 (13)	4 (18)	1 (5.9)
CMC (dorsal)	2 (5.1)	1 (4.55)	1 (5.9)
Education			
High school or less	7 (18)	4 (18)	3 (18)
2-years college	4 (10)	2 (9.1)	2 (12)
4-years college	14 (36)	7 (32)	7 (41)
Post-college graduate degree	14 (36)	9 (41)	5 (29)
Marital status			
Married/Unmarried Couple	32 (82)	17 (77)	15 (88)
Divorced/Separated	4 (10)	3 (14)	1 (5.9)
Widowed	1 (2.6)	1 (4.5)	0 (0)
Single	2 (5.1)	1 (4.5)	1 (5.9)
Race/ethnicity			
White	27 (69)	14 (64)	13 (76)
Black/African American	3 (7.7)	2 (9.1)	1 (5.9)
Latino/Hispanic	5 (13)	3 (14)	3 (18)
Other	4 (10)	3 (14)	2 (12)
Work			
Employed	19 (49)	16 (73)	3 (17)
Retired	18 (46)	6 (27)	12 (71)
Housemaker	1 (2.6)	0 (0)	1 (5.9)
Other	1 (2.6)	0 (0)	1 (5.9)
Pain during injection	7 (3–9)	8 (7–9)	5 (3–7)
Pain after 5 minutes	2 (0–4)	2 (1–4)	1 (0–2)
Pain after 4 hours	3 (1–5)	4 (2–7)	2 (0–4)

Data expressed as n (%, might not add up to 100% due to rounding) or median (interquartile range). Missing variables: *n* = 4 pain after 4 hours.

JSPPPE: Jefferson Scale of Patient Perceptions of Physician Empathy; PCS: Pain Catastrophizing Scale; PHQ: Patient Health Questionnaire; PSEQ: Pain Self-efficacy Questionnaire.

We did not record patients screened for inclusion. As recommended in the CONSORT statement, we did not statistically assess baseline difference between groups. However, there could be concern about potential differences in age, sex and pain catastrophizing (Table 1). Age was the only variable with *p* < 0.10 for pain intensity 4 hours after injection (rho −0.33, *p* = 0.056) (Appendix 1), so we accounted for age as an independent variable in the model. Otherwise, the marginal differences seen in sex and pain catastrophizing, with *p* > 0.10, are less likely to have had a confounding effect on study results.

### Outcome measures

Pain intensity was measured using a non-continuous 0–10 Numerical Rating Scale (NRS-11) during the injection, 5 minutes and 4 hours afterwards. A research assistant called participants to obtain the pain intensity level 4 hours after injection. The NRS-11 is a standard in research to assess pain intensity (Safikhani et al., 2017).

### Other variables

We recorded age, sex, injection site, education, marital status, race, work and the following validated questionnaires: JSPPPE; PCS-4; PSEQ-2; and PHQ-2. We collected JSPPPE, PCS-4, PSEQ-2 and PHQ-2 during the frequentist stage of the study, but after changing to Bayesian, there were not enough participants to meaningfully analyse these measures. We used them to document and confirm the balance between the groups, but they were not otherwise analysed.

The JSPPPE is a five-item measure of the patient’s perception of clinician empathy (Hojat et al., 2010). Patients responded to each item of the survey on a 7-point Likert scale (1 representing strongly disagree, 7 representing strongly agree). The PCS-4 measures unhelpful thoughts and feelings about pain (Walton et al., 2020). Items are scored on a Likert scale of 0–4. The total score is in the range of 0–16, with higher scores representing greater catastrophic thinking. PSEQ-2 measures a helpful approach to pain and includes two items scored on a 7-point Likert scale, which are added to form a total score in the range of 0–12 (Briet et al., 2014). High scores indicate greater self-efficacy. PHQ-2 consists of two items, depressed mood and loss of interest (anhedonia), scored on a scale of 0–3 and added to form a total score in the range of 0–6 (Levis et al., 2020).

### Statistical analysis

Initially this study was set up using a frequentist analysis with an equivalence trial design. The minimal difference in pain intensity that people indicate as relevant is 1 point or more on a scale of 0–10 (Olsen et al., 2017). Based on this knowledge, an a priori power analysis indicated that 118 participants would provide 90% power assuming a 1-point difference and a standard deviation of 1.5 for both groups, with a two-side confidence interval, and alpha set at 0.05.

During the study, we gained experience with the Bayesian trial design and its benefits, such as a continuous analysis as participants complete the study (Teunis et al., 2023). We therefore switched to a Bayesian design, using the same goal of reliably establishing the presence or absence of a 1-point difference in pain intensity between treatments. Specifically, we would stop the trial when there is a 95% probability that the difference in pain intensity directly after injection between treatments is greater than 1.0 point (on a 0–10 scale); and upon repeating this analysis 40 times, at least 36 (90%) attempts need to confirm the greater than 95% probability. The 36 out of 40 (90%) analyses is arbitrarily chosen, as Bayesian analysis is relatively new in healthcare studies, and there are currently no consensus guidelines.

We started our analysis after including 24 participants and repeated it when five more participants were included in the study. We used an uninformative prior for our analysis (difference 0, variance 10,000). We decided on this prior because one previous prospective cohort study found no difference in pain intensity between injections with and without lidocaine after adjusting for mental health (Julka et al., 2012). Another randomized controlled trial reported greater pain intensity injecting corticosteroid with lidocaine and epinephrine comparted to plain corticosteroid (visual analogue pain scale corticosteroid 3.5 vs. with lidocaine and epinephrine 2.0) (Patrinely et al., 2021). However, we were concerned that this difference was based on the epinephrine, which results in a substantially lower pH (lidocaine 1% plain pH 6.09 vs. with epinephrine 1:100,000 pH 4.2).

Bayesian analysis uses a Markov chain Monte Carlo (MCMC) simulation to establish a posterior distribution based on the prior and available data. We used the Metropolis-Hastings sampling algorithm for our MCMC simulation, with 12,500 iterations and a burn-in of 2500. To assess model conversion, we visually inspected trace, histograms, autocorrelation and density plots. The plots of each analysis suggest adequate conversion. To assess MCMC performance, we report the model’s efficiency (conventionally expected to be in the range of 10%–20%, which we obtained for all models).

We used multiple imputations to address missing pain intensity 4 hours after injection (*n* = 4). Pain intensity during injection, after 5 minutes and age were used for imputing. We used a data augmentation algorithm, using MCMC procedures, and calculated the mean of 40 imputed sets.

## Results

### Difference in pain intensity directly after injection

We found lower pain intensity when lidocaine was included in the corticosteroid injection, with a median difference 2.4 points and 95% credible interval (CrI) −4.0 to −0.76. The probability that the addition of lidocaine reduces pain intensity during injection with 1.0 point or more is 95% (Table 2). Upon repeating the analysis 40 times, 36 (90%) sets indicated a greater than 95% probability. In the remaining 10% of repetitions, the probability was close but below 95%.

**Table 2.** Bayesian analysis of the difference in pain intensity between steroid injection with and without lidocaine.

Outcome variable	Median difference^a^	95% credibility interval	Model efficiency^b^	Probability that the difference in pain intensity is >−1.0	Models with >0.95 probability that the difference in pain intensity is >−1.0^c^
Pain during injection	−2.4	−4.0 to −0.76	0.13	0.95	36/40 (90)
Pain after 5 minutes	−1.3	−2.6 to −0.06	0.13	0.70	0/40 (0)
Pain after 4 hours^d^	−1.9	−3.3 to −0.27	0.10	0.87	0/40 (0)

This analysis uses an uninformative prior assuming no difference (0) and a variance of 10,000.

a A negative value indicates lower pain intensity with lidocaine.

b Model efficiency needs to be >0.09 for the analysis to be reliable.

c Forty sets were used for this analysis; data expressed as *n* (%).

d The model for pain after 4 hours accounts for age as a possible confounder.

### Difference in pain intensity 5 minutes after injection

We found lower pain intensity 5 minutes after injection when lidocaine was included in the corticosteroid injection, with a median difference of 1.3 points and 95% CrI −2.6 to −0.06. The probability that the addition of lidocaine reduces pain intensity during injection with 1.0 point or more is 70% (Table 2). Upon repeating the analysis 40 times, 0 sets found a greater than 95% probability.

### Difference in pain intensity 4 hours after injection

We found lower pain intensity 4 hours after injection when lidocaine was included in the corticosteroid injection, with a median difference of 1.9 points and 95% CrI −3.3 to −0.27. The probability that the addition of lidocaine reduces pain intensity during injection with 1.0 point or more is 87% (table 2). Upon repeating the analysis 40 times, 0 sets found a greater than 95% probability.

## Discussion

Somewhat counterintuitively, there are studies documenting less pain associated with corticosteroid injection in the hand and wrist when the injection excludes lidocaine (Patrinely et al., 2021; Zakria et al., 2022). We investigated this in our practice using a Bayesian trial design and found that injecting lidocaine with corticosteroids resulted in lower pain intensity during the procedure compared to corticosteroid injections alone.

The findings of this study should be interpreted in light of some limitations. Despite efforts to conceal the volume of the injection syringe with tape and by preparation from a medical assistant, the sensation of a longer injection for corticosteroid with lidocaine may unblind the surgeon to the treatment being administered. Patients may not notice injection duration, but a surgeon that frequently administers injections may. In addition, the exclusion of non–English-speaking patients may limit the generalizability of our results, although in the setting of the study there were few Spanish-speaking people. The decision to change from a frequentist to a Bayesian trial design after enrolment of several patients was related to the discovery of this alternative trial design during enrolment. The change in the trial after enrolment can introduce bias, but given the nature of Bayesian trials, with frequent ongoing analyses, this may have less impact. The imbalance in the assignments results from the use of a random number generator for randomization. This might have caused the trial to run longer than necessary, but it does not influence the quality of the randomization or blinding. To calculate the difference between groups, Bayesian analysis relies on random simulation. If the analysis is repeated on the same data, the random simulation might provide slightly different results. We chose to repeat our analysis 40 times and aim for a 90% confirmation of a 95% probability of a 1-point difference in pain intensity. We reasoned that a 90% confirmation rate of a 95% probability reflects prudent resource utilization and the fact that adding lidocaine has little downside. For medical decisions with greater potential harm or costs, one might chose both a greater confirmation rate and a greater probability (e.g. 99% probability of a 1 point difference and a 100% confirmation rate).

The finding that lidocaine was associated with lower pain intensity suggests that analgesia has more impact than volume. Our findings are unexpectedly discordant with prior research. For example, in a randomized controlled trial consisting of 73 patients with 110 trigger fingers, the participant rating of post-injection pain intensity was significantly higher for corticosteroid injection with lidocaine and epinephrine compared to corticosteroid with saline (Patrinely et al., 2021). Another randomized double-blind controlled trial found that dermatologic intralesional corticosteroid injections in the arm were more painful with lidocaine and epinephrine compared to corticosteroid with saline (Zakria et al., 2022). Pain associated with corticosteroid injections that include lidocaine and epinephrine may be due to lidocaine with epinephrine being approximately 1000 times more acidic than subcutaneous tissue (Frank and Lalonde, 2012). In a systematic review of 23 studies focusing on non-intravascular injections, it was concluded that increasing the pH of lidocaine (reducing acidity) decreased injection-related pain intensity (Cepeda et al., 2010). In addition, two randomized, double-blind, prospective clinical trials showed reduced injection-related pain intensity when buffering to reduce injection acidity (Bartfield et al., 1993; Lee et al., 2013). However, three studies found that changes in injection pH did not reduce pain intensity from corticosteroid injection with local anaesthetic (Goldfarb et al., 2007; Lewis et al., 2005; Price et al., 1991).

We found a substantial probability that pain intensity is lower 5 minutes and 4 hours after injection, but the probabilities did not reach our predetermined 95% cut-off. A larger sample size would be able to determine if the probability of more or less than a 1-point reduction in pain intensity 5 minutes and 4 hours after injection reaches 95%. Future Bayesian trials interested in answering this question can use our posterior probabilities as priors for their trials, reducing the required sample sizes.

## Appendix 1. Association between age, catastrophic thinking, and sex and outcome variables.

**Table:**

Independent	Age	*p*-value	PCS	*p*-value	Men	Women	*p*-value
Pain during injection	0.0082	0.96	0.20	0.21	6 (4–8)	7 (3–9)	0.50
Pain after 5 minutes	0.036	0.83	0.13	0.44	2 (0–2)	2 (0–4)	0.19
Pain after 4 hours	−0.33	0.056	0.17	0.32	2.5 (0.5–5)	3 (2–5)	0.59

Continuous variables are presented as median (interquartile range).

PCS: Pain Catastrophizing Scale.

## References

[bibr1-17531934241245036] An TW Boone SL Boyer MI Gelberman RH Osei DA Calfee RP. Effect of ice on pain after corticosteroid injection in the hand and wrist: a randomized controlled trial. J Hand Surg Eur Vol. 2016, 41: 984–9. 27402283 10.1177/1753193416657678

[bibr2-17531934241245036] Bartfield JM Ford DT Homer PJ. Buffered versus plain lidocaine for digital nerve blocks. Ann Emerg Med. 1993, 22: 216–9. 8381259 10.1016/s0196-0644(05)80206-9

[bibr3-17531934241245036] Briet JP Bot AGJ Hageman MGJS Menendez ME Mudgal CS Ring DC. The pain self-efficacy questionnaire: validation of an abbreviated two-item questionnaire. Psychosomatics. 2014, 55: 578–85. 25016359 10.1016/j.psym.2014.02.011

[bibr4-17531934241245036] Cepeda MS Tzortzopoulou A Thackrey M Hudcova J Arora Gandhi P Schumann R . Adjusting the pH of lidocaine for reducing pain on injection. Cochrane Database Syst Rev. 2010; 12: CD006581. 10.1002/14651858.CD006581.pub2 21154371

[bibr5-17531934241245036] Frank SG Lalonde DH. How acidic is the lidocaine we are injecting, and how much bicarbonate should we add? Can J Plast Surg. 2012, 20: 71–3. 23730153 10.1177/229255031202000207 PMC3383550

[bibr6-17531934241245036] Goldfarb CA Gelberman RH McKeon K Chia B Boyer MI. Extra-articular steroid injection: early patient response and the incidence of flare reaction. J Hand Surg Am. 2007, 32: 1513–20. 18070637 10.1016/j.jhsa.2007.08.002

[bibr7-17531934241245036] Heise T Nosek L Dellweg S , et al. Impact of injection speed and volume on perceived pain during subcutaneous injections into the abdomen and thigh: a single-centre, randomized controlled trial. Diabetes Obes Metab. 2014, 16: 971–6. 24720741 10.1111/dom.12304

[bibr8-17531934241245036] Hojat M Louis DZ Maxwell K Markham F Wender R Gonnella JS. Patient perceptions of physician empathy, satisfaction with physician, interpersonal trust, and compliance. Int J Med Educ. 2010, 1: 83–7.

[bibr9-17531934241245036] Jørgensen JT Rømsing J Rasmussen M Møller-Sonnergaard J Vang L Musaeus L. Pain assessment of subcutaneous injections. Ann Pharmacother. 1996, 30: 729–32. 8826549 10.1177/106002809603000703

[bibr10-17531934241245036] Julka A Vranceanu A-M Shah AS Peters F Ring D. Predictors of pain during and the day after corticosteroid injection for idiopathic trigger finger. J Hand Surg Am. 2012, 37: 237–42. 22192164 10.1016/j.jhsa.2011.10.055

[bibr11-17531934241245036] Lee HJ Cho YJ Gong HS Rhee SH Park HS Baek GH. The effect of buffered lidocaine in local anesthesia: a prospective, randomized, double-blind study. J Hand Surg Am. 2013, 38: 971–5. 23566722 10.1016/j.jhsa.2013.02.016

[bibr12-17531934241245036] Levis B Sun Y He C , et al. Accuracy of the PHQ-2 alone and in combination with the PHQ-9 for screening to detect major depression: systematic review and meta-analysis. JAMA. 2020, 323: 2290–300. 32515813 10.1001/jama.2020.6504 PMC7284301

[bibr13-17531934241245036] Lewis M Hay EM Paterson SM Croft P. Local steroid injections for tennis elbow: does the pain get worse before it gets better?: Results from a randomized controlled trial. Clin J Pain. 2005, 21: 330–4. 15951651 10.1097/01.ajp.0000125268.40304.b3

[bibr14-17531934241245036] Olsen MF Bjerre E Hansen MD , et al. Pain relief that matters to patients: systematic review of empirical studies assessing the minimum clinically important difference in acute pain. BMC Med. 2017, 15: 35. 28215182 10.1186/s12916-016-0775-3 PMC5317055

[bibr15-17531934241245036] Patrinely JR Johnson SP Drolet BC. Trigger finger corticosteroid injection with and without local anesthetic: a randomized, double-blind controlled trial. Hand (N Y). 2021, 16: 619–23. 31690121 10.1177/1558944719884663 PMC8461190

[bibr16-17531934241245036] Price R Sinclair H Heinrich I Gibson T. Local injection treatment of tennis elbow–hydrocortisone, triamcinolone and lignocaine compared. Br J Rheumatol. 1991, 30: 39–44. 1991216 10.1093/rheumatology/30.1.39

[bibr17-17531934241245036] Safikhani S Gries KS Trudeau JJ , et al. Response scale selection in adult pain measures: results from a literature review. J Patient Rep Outcomes. 2017, 2: 40. 30238085 10.1186/s41687-018-0053-6 PMC6127068

[bibr18-17531934241245036] Saha P Smith M Hasan K. Accuracy of intraarticular injections: blind vs. image guided techniques-a review of literature. J Funct Morphol Kinesiol. 2023, 8: 93. 37489306 10.3390/jfmk8030093 PMC10366715

[bibr19-17531934241245036] Teunis T Samadi A Chen N Ring D. Research methodology: the Bayesian statistical framework and the future of trial design. J Hand Surg Eur Vol. 2023, 48: 593–7. 36756742 10.1177/17531934231152558

[bibr20-17531934241245036] Walton DM Mehta S Seo W MacDermid JC. Creation and validation of the 4-item BriefPCS-chronic through methodological triangulation. Health Qual Life Outcomes. 2020, 18: 124. 32381020 10.1186/s12955-020-01346-8 PMC7204020

[bibr21-17531934241245036] Zakria D Patrinely JR Dewan AK , et al. Intralesional corticosteroid injections are less painful without local anesthetic: a double-blind, randomized controlled trial. J Dermatolog Treat. 2022, 33: 2034–7. 33760691 10.1080/09546634.2021.1906842

[bibr22-17531934241245036] Zijlstra E Jahnke J Fischer A Kapitza C Forst T. Impact of injection speed, volume, and site on pain sensation. J Diabetes Sci Technol. 2018, 12: 163–8. 28990437 10.1177/1932296817735121 PMC5761988

